# HERV-W Envelope Triggers Abnormal Dopaminergic Neuron Process through DRD2/PP2A/AKT1/GSK3 for Schizophrenia Risk

**DOI:** 10.3390/v14010145

**Published:** 2022-01-14

**Authors:** Qiujin Yan, Xiulin Wu, Ping Zhou, Yan Zhou, Xuhang Li, Zhongchun Liu, Huawei Tan, Wei Yao, Yaru Xia, Fan Zhu

**Affiliations:** 1 State Key Laboratory of Virology, Department of Medical Microbiology, School of Basic Medical Sciences, Wuhan University, Wuhan 430071, China; qingqiu@whu.edu.cn (Q.Y.); 2017203010009@whu.edu.cn (X.W.); zhouping@whu.edu.cn (P.Z.); zhouyan008007009@163.com (Y.Z.); lixuhangsiia@126.com (X.L.); yaowei1234567@sina.com (W.Y.); xyr516549594@163.com (Y.X.); 2 Department of Psychiatry, Renmin Hospital of Wuhan University, Wuhan 430060, China; zcliu6@whu.edu.cn (Z.L.); tomybeckham@126.com (H.T.); 3 Hubei Province Key Laboratory of Allergy & Immunology, Wuhan University, Wuhan 430071, China

**Keywords:** human endogenous retrovirus, HERV-W ENV, schizophrenia, dopamine receptor D2, dopaminergic systems, sodium influx

## Abstract

An increasing number of studies have begun considering human endogenous retroviruses (*HERVs*) as potential pathogenic phenomena. Our previous research suggests that HERV-W Envelope (HERV-W ENV), a HERV-W family envelope protein, is elevated in schizophrenia patients and contributes to the pathophysiology of schizophrenia. The dopamine (DA) hypothesis is the cornerstone in research and clinical practice related to schizophrenia. Here, we found that the concentration of DA and the expression of DA receptor D2 (DRD2) were significantly higher in schizophrenia patients than in healthy individuals. Intriguingly, there was a positive correlation between HERV-W ENV and DA concentration. Depth analyses showed that there was a marked consistency between HERV-W ENV and DRD2 in schizophrenia. Studies in vitro indicated that HERV-W ENV could increase the DA concentration by regulating DA metabolism and induce the expression of DRD2. Co-IP assays and laser confocal scanning microscopy indicated cellular colocalization and a direct interaction between DRD2 and HERV-W ENV. Additionally, HERV-W ENV caused structural and functional abnormalities of DA neurons. Further studies showed that HERV-W ENV could trigger the PP2A/AKT1/GSK3 pathway via DRD2. A whole-cell patch-clamp analysis suggested that HERV-W ENV enhanced sodium influx through DRD2. In conclusion, we uncovered a relationship between HERV-W ENV and the dopaminergic system in the DA neurons. Considering that GNbAC1, a selective monoclonal antibody to the MSRV-specific epitope, has been promised as a therapy for treating type 1 diabetes and multiple sclerosis (MS) in clinical trials, understanding the precise function of HERV-W ENV in the dopaminergic system may provide new insights into the treatment of schizophrenia.

## 1. Introduction

Endogenous retroviruses (*ERVs*) are present in the genomes of all vertebrates, as well as in some invertebrates [1]. Since being discovered in 1981 [2], human endogenous retroviruses (*HERVs*) constitute about 8% of the human genome [3]. Although once ascribed to “junk DNA”, many studies suggest that *HERVs* could regulate pleiotrophin in the placenta, apolipoprotein C1 in the liver, and *β-amylase* in the salivary glands [4]. Abnormal expression of HERV-W ENV, a HERV-W envelope protein, has been intensively investigated for its putative role in several diseases, such as bladder cancer [5], multiple sclerosis (MS) [6], hepatocellular carcinoma [7], type 1 diabetes [8], and bipolar disorder [9].

The HERV-W family draws the attention of most researchers who study *HERVs* because of its founder member, multiple sclerosis-associated retroviruses (*MSRV*), a complete virus with extracellular virions. Additionally, its envelope protein (also called Syncytin-1), located on human chromosome 7q21-22, has pivotal physiological functions during early pregnancy [3]. Quite a few studies suggest that HERV-W ENV constitute a possible link between genetic components and environmental factors in schizophrenia [10,11,12,13,14,15]. Investigation into HERV-W ENV will further our understanding of the molecular basis of schizophrenia pathogenesis.

Schizophrenia, a severe and chronic mental illness characterized by abnormal behavior, strange speech, and decreased ability to understand reality [16], is among the top ten leading causes of disability worldwide [17]. The etiology of schizophrenia is multifactorial. There are quite a few hypotheses [18], and the DA hypothesis is one of the most enduring ideas in psychiatry [19]. It suggests that a dysregulated DA system contributes to the development of schizophrenia. This hypothesis is supported by the fact that large numbers of antipsychotics have DA-receptor antagonistic effects. Currently, the pathogenesis of schizophrenia is still unclear. Moreover, there is no report on the link between HERV-W ENV and the DA system.

*ARRB2* (arrestin beta 2) is identified as an important mediator between DRD2 and the serine/threonine protein kinase (AKT) signal cascade. This implicates *ARRB2* as a potential pharmacological target for dopamine-related psychiatric disorders [20]. Clinical data indicate that *ARRB2* is associated with Chinese schizophrenia patients [21]. Numerous studies have provided direct evidence for the AKT/GSK3 pathway in schizophrenia [22,23,24]. PP2A mediates dopaminergic neurotransmission. Consistent with the ARRB2/PP2A complex formation, the ARRB2/PP2A/AKT1/GSK3 signal pathway facilitates the dephosphorylation and deactivation of AKT, resulting in the activation of GSK3, and may be an attractive potential factor in the DA hypothesis of schizophrenia [25].

In this paper, we found a high expression of DA receptor D2 (DRD2) and a significant consistency between HERV-W ENV and DRD2 in schizophrenia patients. Intriguingly, DA concentration was notably higher in schizophrenia patients than in healthy persons. Moreover, DA concentration was higher in HERV-W ENV-positive schizophrenia patients than that in HERV-W ENV-negative patients. In DA neurons, DA concentration increased through enhanced DA synthesis and transport by HERV-W ENV. The DA system was activated by the upregulated DRD2 and synaptic proteins. In the DA neurons, a series of experiments showed that HERV-W ENV increased DA concentration by upregulating tyrosine hydroxylase (TH) and enhanced DA release through regulating the DA transporter (DAT) and solute carrier family 18 member A2 (VMAT2). Further studies found that HERV-W ENV activated the dopaminergic system by upregulating the DRD2 and synaptic proteins. We observed that DRD2 and HERV-W ENV directly interacted or colocalized in the membrane of DA neurons. Additionally, HERV-W ENV increased the expression of several synaptic proteins involved in neurotransmitter release and synaptic plasticity. In-depth research revealed that DRD2 was essential for the ARRB2/PP2A/AKT1/GSK3 pathway activated by HERV-W ENV. Thus, on this basis, our results suggested that HERV-W ENV contributed to the abnormal DA neuron process and had the potential to evolve as a novel potential therapeutic target for schizophrenia.

## 2. Materials and Methods

### 2.1. Bioinformatics

NCBI-GEO is a free public database of microarray/gene profiles. We obtained the gene expression profile of GSE21935, which contains 23 schizophrenia and 19 healthy controls [26]. Microarray data were based on the GPL570 Platform ([HG-U133_Plus_2] Affymetrix Human Genome U133 Plus 2.0 Array). Differentially expressed genes (DEGs) between schizophrenia and healthy controls were identified via the GEO2R online tools [27] with |log FC| > 2 and *p*-value < 0.05. The DEGs with log FC > 0 were considered as upregulated genes. Gene ontology analysis (GO) [28] and the Kyoto Encyclopedia of Genes and Genomes (KEGG) [29] were applied by DAVID [30], which is an online tool designed to identify gene or protein functions. We utilized DAVID to visualize the DEGs’ enrichment of biological process (BP), molecular function (MF), cell component (CC), and pathways (*p* < 0.05). GSE25673, a dataset from schizophrenic hiPSC (human induced pluripotent stem cells)-derived neurons [31], was also used to analyze the expression of GSK3β and the DA receptors.

### 2.2. Clinical Samples and Ethical Considerations

All patients were from the Renmin Hospital, Wuhan University. Blood samples were recruited from 57 patients with recent-onset schizophrenia according to the *Diagnostic and Statistical Manual of Mental Disorders* Fifth Edition (DSM-V) on an empty stomach at 8:00 a.m. None of the patients had a history of neuroleptic treatment. They had no manifestation of acute infectious, inflammatory, or neurological diseases. Sixty-eight healthy controls were healthy donors obtained from the health check-up, also from the Renmin Hospital of Wuhan University. Additionally, they were absent of any illness or disease. The power analysis of the sample sizes showed that the power calculation reached 0.99 (alpha = 0.05).

Blood sample collections were performed under the principles of the Declaration of Helsinki and approved by the Ethics Committee of the School of Basic Medicine of Wuhan University (grant #06R-1366). The research was sponsored according to the International Ethical Guidelines for Biomedical Research involving Human Subjects (CIOMS).

### 2.3. The Construction of Plasmids

The plasmids, *pCMV-HERV-W ENV* [10] and *pEGFP-HERV-W ENV-TM* [12], were obtained as previously described. The *pENTER-DRD2* plasmid was purchased from Vigene Biosciences. The promoters of human *DRD2* or *VMAT2*, predicted by UCSC, were cloned into luciferase reporter vector *pGL3-basic*. The *pXJ40-HA-HERV-W ENV* and the *pIRES2-EGFP-HERV-W ENV* were constructed according to the manufacturer’s instruction (Invitrogen, Catalog nos. V790-20, Carlsbad, CA, USA).

### 2.4. Cell Culture and Transfection

The human dopaminergic neuroblastoma cell line (SH-SY5Y) was purchased from ATCC. The cell line was cultured in Dulbecco’s modified Eagle’s medium (DMEM) (GIBCO, Newyork, CA, USA) supplemented with 10% heat-inactivated fetal bovine serum (FBS) (GIBCO, Newyork, CA, USA) and 1% penicillin/streptomycin. Primary midbrain DA neurons from neonatal *Sprague Dawley* (SD) rats, regardless of gender, following the National Institutes of Health’s Guide for the Care and Use of Laboratory Animals, was prepared as previously described [32]. Animal experiments were approved by the Animal Ethics Committee of Wuhan University Center for Animal Experiment/A3 Laboratory, Wuhan University. Primary neurons were maintained in DMEM/F12 supplemented with 10% FBS, 1% Gluta-max (Invitrogen, 35050, Carlsbad, CA, USA), 1% Sodium Pyruvate (Invitrogen, 11360070, Carlsbad, CA, USA), 1% NEAA (Invitrogen, 11140, Carlsbad, CA, USA), and 1% penicillin/streptomycin at 37 °C with 5% CO_2_. Immunofluorescence of TH (Abcam, ab137869, 1:100, Cambridge, MA, USA) and TUJ1 (beta III Tubulin) (Abcam, ab78078, 1:150, Cambridge, MA, USA) served to identify primary DA neurons (Figure S1). DA neurons (including SH-SY5Y and the primary neurons) were transfected with LipofectamineTM2000 (Invitrogen, 11668030, Carlsbad, CA, USA) according to the manufacturer’s instructions.

### 2.5. ELISA

The DA concentrations in the serum, culture supernatants, and intracellular fluid were determined using commercially available standard sandwich ELISA kits (Elabscience, E-EL-0046c, Wuhan, China). ELISA kits for DRD1 (DLdevelop, DLR-DRD1-Hu, Wuxi, China), DRD2 (Wallner, 15560, Wuhan, China), and HERV-W ENV (Wallner, 13282, Wuhan, China) were used according to the kit manufacturers’ instructions. The absorbance measured at a 450 nm wavelength by an ELISA reader (Multiskan FC 357, Thermo, Waltham, MA, USA), and the concentrations were calculated based on the standard curve. Data represent the mean of the results from each sample tested in duplicate.

### 2.6. Cellular Immunofluorescence

Cells were fixed in 4% paraformaldehyde and penetrated with 0.5% Triton X-100 in PBS (pH 7.4). The cells were incubated with the diluted primary antibody at 4 °C overnight. After washing, the cells were incubated with Cy3-labeled rabbit anti-mouse IgG antibody (Abcam, ab97035, Cambridge, MA, USA) at 37 °C for 1 h. Then, the anti-fluorescence quenching sealing solution (including Hoechst 33342, which is responsible for nuclear staining) (Beyotime, P0133, Shanghai, China) was added, and cells were imaged by fluorescence microscopy. The positive cells appeared yellow due to the overlap of red and green colors.

### 2.7. Real-Time Quantitative Reverse Transcriptase-PCR

Total RNA was isolated from cells using Trizol Reagent (Invitrogen, 15596018, Carlsbad, CA, USA). After treatment with DNase *I* (Thermo, EN0521, Waltham, MA, USA) to remove genomic DNA contaminations from total RNA, first-strand cDNA synthesis was performed using an MMLV Reverse Transcriptase 1st-Strand cDNA Synthesis Kit (Invitrogen, 18091200, Carlsbad, CA, USA) following the manufacturer’s protocol. Real-time quantitative PCR was performed on the iCycler system (C1000, Bio-Rad, Hercules, CA, USA) in a final volume of 25 µL using the SYBR Green master mixture. *GAPDH* was used as an endogenous control to normalize the gene expression data.

The primers were designed based on sequences obtained from the NCBI database using Primer 5 software and listed in the Tables S1 and S2.

### 2.8. Western Blotting Analysis

Protein was extracted from cultured cells lysed with mammalian protein extraction reagent (Thermo, 78505, Waltham, MA, USA) supplemented with protease inhibitor mixture (Sigma, P8340, Steinheim, Germany), and protein concentrations were measured using a BCA protein Assay kit (Thermo, 23250, Waltham, MA, USA). Western blotting was performed using the standard method. All antibodies were purchased from Abcam (Table S3). Corresponding secondary antibodies were used. Protein bands detected by the antibodies were visualized with ECL reagents (Millipore, 34080, Billerica, MA, USA) in a Tanon 5200 MultiImage System. The levels of protein were normalized to GAPDH, one of the housekeeping genes.

### 2.9. Luciferase Assay

SH-SY5Y cells were co-transfected with either the *pGL3-VMAT2* or *pGL3-DRD2* promoter, plasmid *pCMV-HERV-W ENV*, or an empty plasmid *pCMV*. The transfection efficiency was normalized against the Renilla luciferase vector *phRL-TK*. Forty-eight hours after transfection, luciferase activities were measured using the dual-luciferase reporter assay system (Promega, E1960, Madison, WI, USA) according to the manufacturer’s protocol.

### 2.10. FM 1-43 Staining of Synaptic Vesicles

The only known function of synaptic vesicles (SVs) is the neurotransmitter store and release. A fluorescent dye, FM 1-43 was used to detect the number and distribution of SVs [33]. After 48 h of transfection, the cells were washed twice with pre-warmed Krebs-Ringer buffer (Abbreviation: KRB; 130 mmol/L NaCl, 3.6 mmol/L KCl, 0.5 mmol/L NaH_2_PO_4_, 0.5 mmol/L MgSO_4_, 10 mmol/L HEPES, 2.0 mmol/L NaHCO_3_, 1.5 mmol/L CaCl_2_, 5.5 mmol/L Glucose). Then, 10 µmol/L FM 1-43 (Biotium, Cat. 70020), diluted with K+-KRB (KCl concentration in the KRB was adjusted to 60 mmol/L) was added for 60 s at 37 °C. The cells were washed once with PBS (pH 7.4) and fixed with 4% paraformaldehyde in PBS for 20 min. After washing three times by PBS (pH 7.4), the slides were sealed with the anti-fluorescence quencher (Beyotime, P0133, Shanghai, China), and the images were observed under a confocal fluorescence microscope (Leica-LCS-SP8-STED, Leica Microsystems, Wetzlar, Germany).

### 2.11. Confocal Laser Scanning Microscopy

Confocal laser scanning microscopy was performed as described previously [12] using a Leica-LCS-SP8-STED (Leica Microsystems, Wetzlar, Germany). HEK293T and SH-SY5Y cells were co-transfected with *pEGFP-HERV-W ENV-TM* and *pENTER-DRD2*. Forty-eight hours after transfection, dual-color STED images were recorded with an HCX PL APO 63⁄/1.40–0.60 oil objective lens. The fluorescences were excited at 561 nm (Red), 488 nm (Green), and 405 nm (Blue). We analyzed two-color STED images using Pearson’s correlation coefficient as well as the overlap and colocalization coefficients introduced by Manders 2013 et al. [34].

### 2.12. Co-Immunoprecipitation Assay

Co-immunoprecipitation was performed as described previously [12]. Anti-flag (Abcam, ab205606, 1:1000, Cambridge, MA, USA), anti-HA (Abcam, ab1424, 1:1000, Cambridge, MA, USA), and mouse IgG (Abcam, ab179693, 1:500, Cambridge, MA, USA) primary antibodies were purchased from Abcam; Protein A/G Agarose was obtained from Thermo Scientific (Catalog: 20421, Waltham, MA, USA). The immunoprecipitated proteins were analyzed by Western blotting.

### 2.13. PP2A Activity Assay

The activity of PP2A was determined using the Ser/Thr phosphatase assay system (Promega, V2460, Madison, WI, USA) according to the manufacturer’s protocol [35]. The optical density values of the samples were read at 600 nm on a Thermo Scientific Multiskan FC 357 (Thermo, Waltham, MA, USA). The PP2A activity was calculated as the ratio of free phosphate released from the pre-phosphorylated peptide and normalized to the basal levels (sample without phosphopeptides).

### 2.14. Pharmacologic Treatment

LB-100 (MedChem Express, HY-18597, Shanghai, China), a water-soluble PP2A inhibitor, was used to reduce PP2A activity. Droperidol (MedChem Express, HY-B1240, Shanghai, China), a short-acting butyrophenone that acts as a potent DRD2 antagonist, was used to block DRD2 in DA neurons. Cells were treated with 1 mmol/L LB-100 or 10 μmol/L Droperidol in a humidified incubator with 5% CO_2_ for 24 h at 37 °C, followed by Western blotting and electrophysiology.

The human *DRD2-siRNA* (*si-DRD2*) and negative control (*si-NC*) were purchased from Ribobio (Guangzhou, China). The target sequence of *si-DRD2* was GCGAGCATCCTGAACTTGT. After being transfected for 24 h, the cells were used for Western blotting.

### 2.15. Electrophysiology

Sodium current plays a major role in the action potential in midbrain DA neurons [36]. Whole-cell patch-clamp recording is a powerful tool to study sodium current directly. To measure Na+ channel currents, pipettes were filled with a pipette solution containing: 130 mmol/L aspartic acid, 130 mmol/L cesium hydroxide, 5 mmol/L EGTA, 2 mmol/L disodium-ATP, 0.1 mmol/L sodium-GTP, 10 mmol/L HEPES, and 5 mmol/L MgCl_2_ (pH adjusted to 7.2 with 1 M CsOH). The cells were filled with a bath solution consisting of 135 mmol/L NaCl, 5 mmol/L KCl, 10 mmol/L HEPES, 4 mmol/L MgCl_2_, 5 mmol/L EGTA, 10 mmol/L tetraethylammoniums, and 10 mmol/L glucose (pH adjusted to 7.4 with 1 M NaOH). Furthermore, the Na+ channel currents were elicited by 20 ms step depolarizations to −20 mV from a holding potential of either −100 mV or −70 mV every 10 s. Currents were recorded by an EPC-10 patch-clamp amplifier (Heka Electronic, GmbH, Germany). Data were initially analyzed with Patchmaster software (Heka Electronic, GmbH, Germany).

### 2.16. Statistical Analysis

Data were expressed as mean ± SD from at least three independent experiments. For the clinical data, the median analysis was performed. Additionally, the relationship between HERV-W ENV and DRD2 was analyzed with the correlation analysis. Statistical analysis performed using SPSS13.0. Two-way analysis of variance and Student’s *t*-tests were used to analyze the data. The minimal level of statistical significance was set at *p* < 0.05. All the experiments were performed blindly.

## 3. Results

### 3.1. Bioinformatics Analysis Demonstrates That DRD1, DRD2, and GSK3 Are Implicated in Schizophrenia

**Figure 1:**
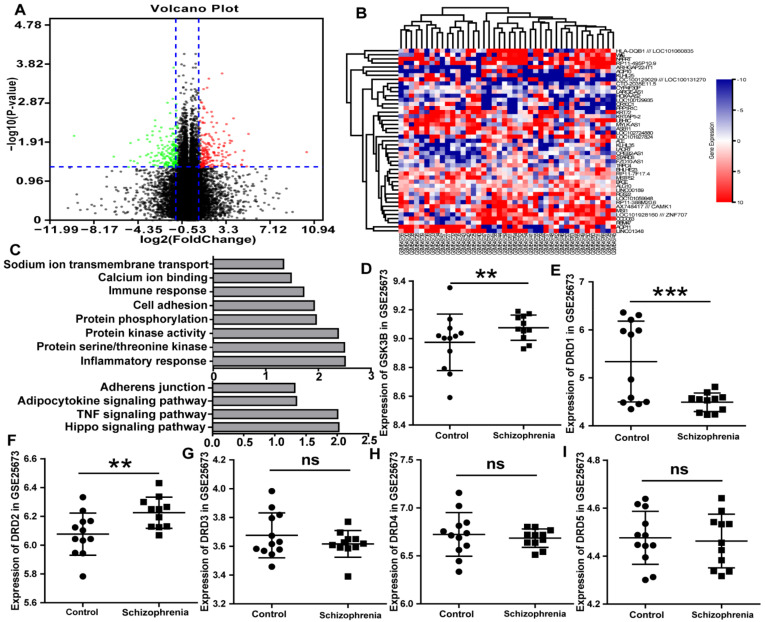
*GSK3, DRD1*, and *DRD2* are highly expressed in schizophrenia according to bioinformatics. (**A**) the volcano plot of each gene expression profile data and 255 differentially expressed genes identified using R software. (**B**) The heat map of 255 DEGs. (**C**) GO and KEGG analyses of the DEGs suggested an enrichment in the inflammatory response, protein serine/threonine kinase activity, protein kinase activity, and Hippo signaling pathway. (**D**) The copy number of GSK3β in schizophrenia GSE25673 was higher than in the control. (**E**) The level of *DRD1* in schizophrenia was less than that in the control in GSE25673. (**F**) The level of *DRD2* in schizophrenia was higher than that in the control in GSE25673. (**G**–**I**) There was no difference in *DRD3, DRD4,* and *DRD5* in schizophrenia and the control. ns *p* > 0.05, ** *p* < 0.01, *** *p* < 0.001.

Via GEO2R online tools, the microarray output showed that there were 255 DEGs, including 108 downregulated genes (log FC < −2) and 147 upregulated genes (log FC > 2) (Figure 1A,B) in schizophrenic patients. These DEGs were mainly enriched in several biological pathways, including the regulation of protein serine/threonine kinase activity, protein kinase activity, protein phosphorylation, and sodium ion transmembrane transport (Figure 1C and Table S4). Additionally, the DEGs analyzed by the KEGG (Figure 1C and Table S5) were focused on the Hippo signaling pathway. Additionally, the Hippo signaling pathway was regulated by protein phosphorylation and protein serine/threonine kinase. Meanwhile, serine/threonine kinase activity could lead to phosphorylation of GSK3. Data from the GSE25673 dataset indicated that *GSK3β* was significantly higher in schizophrenia than that in control (*p* ≤ 0.01; Figure 1D).

DA receptor subtypes are divided into two major subclasses: the D1-like (including *DRD1* and *DRD5*) and D2-like (including *DRD2*, *DRD3*, and *DRD4*) [37]. The level of *DRD1* in schizophrenia was less than in the control in GSE25673 (*p* < 0.001; Figure 1E). Additionally, the level of *DRD2* in schizophrenia was higher than in the control in GSE25673 (*p* < 0.01; Figure 1F). Moreover, there were differences in the levels of *DRD3*, *DRD4*, and *DRD5* in schizophrenia and the control, but the difference did not reach the statistical level in GSE25673 (Figure 1G–I). In conclusion, the *DRD1*, *DRD2*, and *GSK3* were expressed differentially between the schizophrenia patients and the healthy controls.

### 3.2. Correlation and Consistency among DA, HERV-W ENV, and DRD2 in First-Episode Schizophrenia Patients

DA is a key neurotransmitter involved in the pathology of schizophrenia [38]. There is little research reporting serum DA level in schizophrenia. In this paper, we detected the levels of serum DA in both the schizophrenia patients and controls. There were no differences in gender, age, body mass index, education, smoking history between the schizophrenia patients and the healthy controls (Table S6).

**Figure 2:**
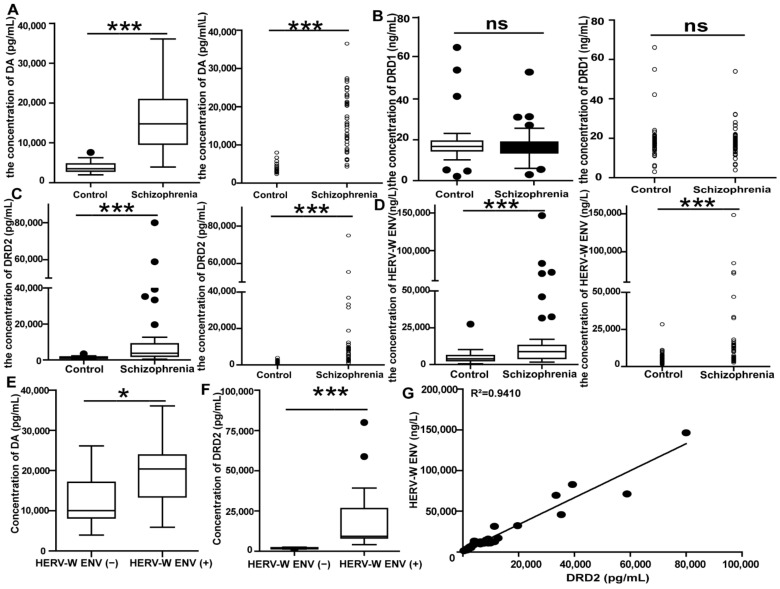
The correlation and consistency between DRD2 and HERV-W ENV in schizophrenia patients: (**A**–**D**) The concentration of DA, DRD1, DRD2, and HERV-W ENV in the control groups (*n* = 68) and the schizophrenia patients (*n* = 57). (**E**,**F**) The concentration of DA and DRD2 in the HERV-W ENV-negative schizophrenia (HERV-W ENV (−), *n* = 25) and the HERV-W ENV-positive schizophrenia (HERV-W ENV (+), *n* = 32) groups. (**G**) Correlation between HERV-W ENV antigenemia and DRD2 levels in schizophrenia patients. *X*-axis: DRD2 value for each schizophrenia patient. *Y*-axis: the concentration for the HERV-W ENV antigen with the HERV-W ENV antibody, obtained by enzyme immunosorbent assay (ELISA). The line represents the calculated “best-fit” equation of the values within the boxed area, with the correlation value indicated on the top (R^2^). ns *p* > 0.05, * *p* < 0.05, *** *p* < 0.001.

The serum DA levels in the 57 schizophrenia patients were substantially higher than those in the 68 healthy individuals (*p* < 0.001 after outlier removal; Figure 2A and Table S7).

DRD1 was detected and showed no alteration between the schizophrenia and the healthy controls (*p* > 0.05; Figure 2B and Table S8). DRD2 is the central receptor for most antipsychotic drugs [18,19]. There have been numerous studies on *DRD2* mRNA levels in schizophrenia, but the results are inconsistent [39,40,41]. Additionally, there is currently no report on detecting the protein levels of serum DRD2 in schizophrenia. As with DA, the expression of DRD2 was also significantly higher in schizophrenia patients than in controls (*p* < 0.001 after outlier removal; Figure 2C and Table S9).

Consistent with our previous study [12], we observed a high level of HERV-W ENV in patients (*p* < 0.001 after outlier removal; Figure 2D and Table S10). Hence, we found a positive correlation between HERV-W ENV and DA (Figure 2E). The median analysis demonstrated that the levels of DA were higher in HERV-W ENV-positive patients (HERV-W ENV (+), concentration ≥ 5415.1 ng/L) than that in HERV-W ENV-negative ones (HERV-W ENV (−), concentration < 5415.1 ng/L) (*p* < 0.05; Figure 2E and Table S11). Of the 32 HERV-W ENV (+), 29 patients (90.63%) were DA positive (concentration ≥ 5000.4 pg/mL), which was greater than 18/25 (72%) in the HERV-W ENV-negative patients (Table 1). Further analysis showed that HERV-W ENV tends to be positively related to DA concentrations in the schizophrenia patients and the healthy groups (Table 1). Moreover, a Spearman analysis also suggested that there were significant positive correlations between the levels of DA and HERV-W ENV (Table 2) (r = 0.426, *p* < 0.01).

Meanwhile, the uniformity and correlation properties between HERV-W ENV and DRD2 were analyzed. The expression of HERV-W ENV was consistent with DRD2 in schizophrenia patients. Of the 32 HERV-W ENV (+), 32 (100%) were DRD2 positive (concentration ≥ 1694.5 pg/mL), which was greater than 17/25 (68%) in the HERV-W ENV-negative patients. The DRD2 median in HERV-W ENV (+) was much higher than that in HERV-W ENV (−) (*p* < 0.0001; Figure 2F and Table S12). Moreover, HERV-W ENV and DRD2 tend to be expressed simultaneously. The rate was as high as 70.18%, indicating a marked consistency between HERV-W ENV and DRD2 expressions in the schizophrenia group. Furthermore, there was also a high frequency of coexistence (77.94%) between HERV-W ENV and DRD2 expressions in the normal control. Additionally, Spearman analysis also suggested that there were significant positive correlations between the levels of HERV-W ENV and DRD2 (r = 0.96, *p* < 0.001). Linear regression indicated a visual representation of the robust correlation between HERV-W ENV and DRD2 (R^2^ = 0.9410) in schizophrenia patients (Figure 2G). In summary, this evidence suggested that DA, DRD2, and HERV-W ENV, which were all markedly upregulated, showed a marked consistency in schizophrenia patients.

### 3.3. HERV-W ENV Promotes the DA Concentration via Regulating DA Metabolism, Including Accelerating TH and DAT

**Figure 3:**
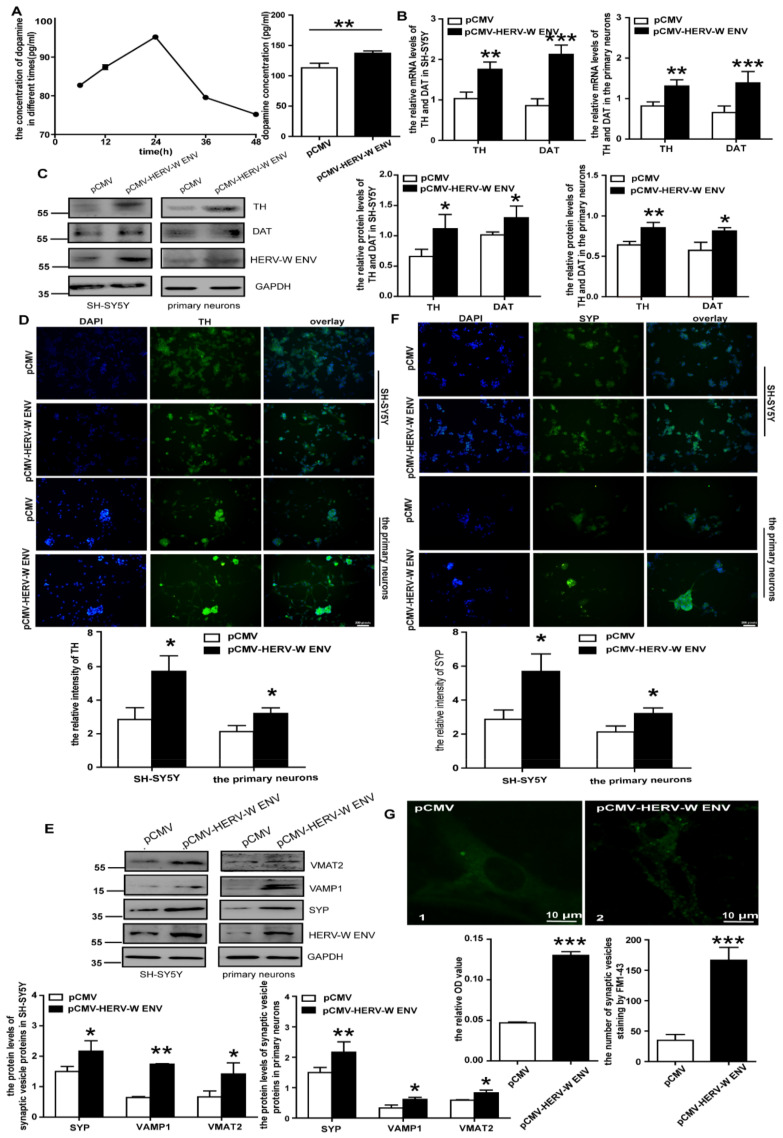
HERV-W ENV facilitates the synthesis and transport of DA: (**A**) DA concentration in the culture supernatants of SH-SY5Y cells reached the highest peak 24 h after transfection and the concentration in the HERV-W ENV overexpressed group was greater than the control. (**B**) The mRNA levels of TH and DAT in the HERV-W ENV overexpression group were higher than the control group in the DA neurons. (**C**) The protein level of TH and DAT in the HERV-W ENV overexpression group was higher than the control group in the DA neurons. (**D**) Cellular immunofluorescence assays of TH in the DA neurons. (**E**) Representative Western blots for the synaptic vesicle-related proteins and GAPDH in the DA neurons. (**F**) SYP expressed higher in the HERV-W ENV overexpression group by cellular immunofluorescence. (**G**) STED images of FM 1-43 in the primary neurons between the *pCMV* group (1) and *pCMV-HERV-W ENV* group (2). The OD value and number of SVs increased after HERV-W ENV overexpression. * *p* < 0.05, ** *p* < 0.01, *** *p* < 0.001.

DA is a neurotransmitter in DA neurons. Therefore, we used the human dopaminergic neuroblastoma cell line SH-SY5Y [42] and rat primary DA neurons to study the causal relationship between HERV-W ENV and the DA system in DA neurons. DA concentration was significantly high, and it changed with time after being transfected with *HERV-W ENV* within 48 h (*p* < 0.01; Figure 3A).

DA metabolism, including the synthesis, storage, release, recycling, and degradation in the presynaptic terminal and synaptic cleft, contributes to DA levels [43]. TH is a rate-limiting enzyme responsible and essential for DA synthesis [44]. Typically, DA is recycled back into the transmitting neuron by a specialized protein called DAT [45]. Intriguingly, HERV-W ENV significantly increased TH and DAT mRNA and protein levels (Figure 3B,C). Meanwhile, immunofluorescent staining proved that HERV-W ENV induced an increased TH protein expression in DA neurons (Figure 3D). These data suggested HERV-W ENV could increase DA concentration by upregulating the expression of TH and DAT.

### 3.4. HERV-W ENV Enhances the Transport of DA

After synthesis, DA is stored in synaptic vesicles (SVs) after uptake by VMAT2 [46]. Importantly, HERV-W ENV could prominently upregulate mRNA (Figure S2A,B) and protein (Figure 3E) levels of VMAT2 in DA neurons. The dual-luciferase assay demonstrated that HERV-W ENV could reinforce the activity of the *VMAT2* promoter (Figure S2C), indicating that HERV-W ENV played a regulatory role at the transcriptional level.

SYP (Synaptophysin) and VAMP1 (vesicle associated membrane protein 1) are essential proteins that present on all SVs for vesicle functions [47]. The results suggested that HERV-W ENV significantly upregulated the mRNA (Figure S2A,B) and protein (Figure 3E) levels of SYP and VAMP1 in the DA neurons. Meanwhile, immunofluorescence showed that SYP staining increased after the transfection of *HERV-W ENV* (Figure 3F).

Except for VMAT2, SVs also represent a decisive point of regulation in DA synaptic transmission [48]. FM1-43 staining indicated that HERV-W ENV significantly increased the total number of SVs and the number of SVs that travelled down the axon to the distal axon terminal in the primary DA neurons (Figure 3G). In addition, OD value, which could reflect the change in absorbance caused by altering the staining number of vesicles, also showed that HERV-W ENV remarkably increased the staining number of vesicles (Figure 3G).

These data indicated that HERV-W ENV could enhance the transport of DA by upregulating the levels of SV proteins.

### 3.5. HERV-W ENV Directly Interacts with DRD2 and Increases the Expression of DRD2

**Figure 4:**
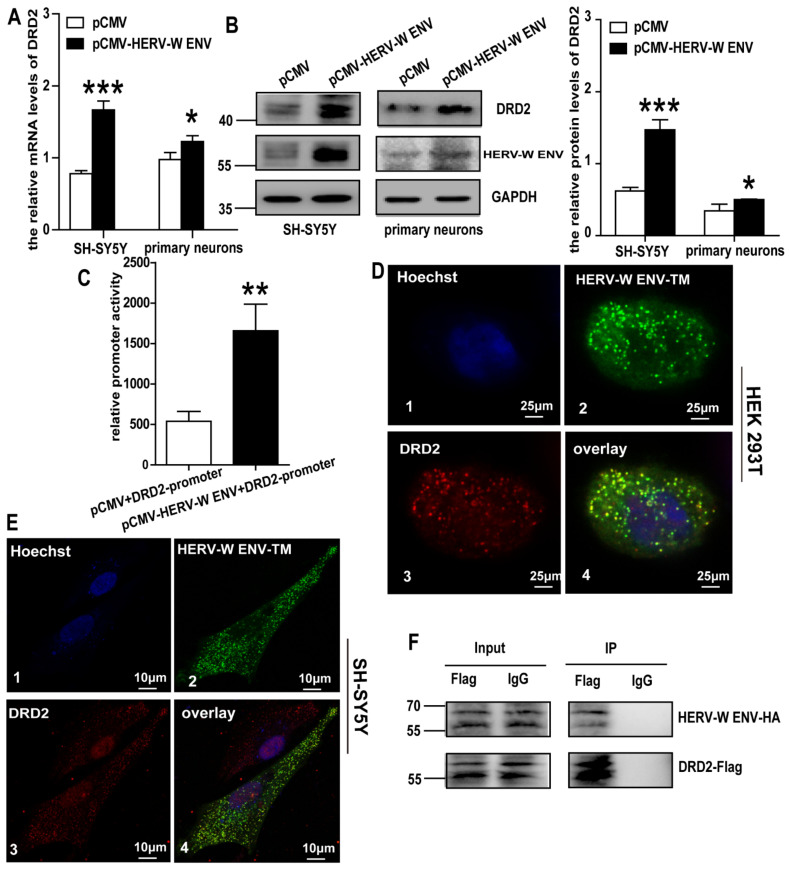
HERV-W ENV increases DRD2 expression by colocalizing and interacting with it: (**A**) the mRNA level of DRD2 in the HERV-W ENV overexpression group was higher than the control group in the DA neurons. (**B**) The protein level of DRD2 in the HERV-W ENV overexpression group was higher than the control group in the DA neurons. (**C**) Luciferase assays of *pGL3-DRD2* promoter co-transfected with *pCMV-HERV-W ENV* or control vector in SH-SY5Y cells. (**D**) Three-color STED images of Hoechst, which specifically stain the nuclei (D1), HERV-W ENV-TM (D2), and DRD2 (D3) in HEK293T cells (D4). Three-color overlay STED images of Hoechst, *pEGFP-HERV-W ENV-TM*, and *pENTER-DRD2* in HEK293T cells. (**E**) Three-color STED images performed as in Figure 4D in SH-SY5Y cells. (**F**) Co-IP results shown in the DRD2-Flag-IP group could show the HERV-W ENV-HA band but could not show such in the negative control, which demonstrated the interactions between each other. * *p* < 0.05, ** *p* < 0.01, *** *p* < 0.001.

DRD2 regulates the levels of DA in the synaptic cleft [43]. Our clinical results showed a significant positive correlation and a robust consistency between HERV-W ENV and DRD2 in schizophrenia patients. Intriguingly, we found that HERV-W ENV potentiated the expression of DRD2 in both mRNA and protein levels (Figure 4A,B) in the DA neurons. The Dual-luciferase assay suggested that HERV-W ENV significantly stimulated *DRD2* promoter activity (Figure 4C).

HERV-W ENV has a transmembrane (TM) subunit that acts as a viral fusion protein. The confocal result indicated that HERV-W ENV-TM appeared to colocalize with pENTER-DRD2 in both HEK293T (crosscorrelation coefficient is 0.83) and SH-SY5Y cells (crosscorrelation coefficient is 0.59) (Figure 4D,E). Additionally, the Co-IP assay showed that specific bands were detected for HA-tagged proteins pulled down with the Flag-tagged antibody, which implied that HERV-W ENV directly interacted with DRD2 (Figure 4F). These results indicated that HERV-W ENV induced the expression of DRD2 by direct interaction with DRD2.

### 3.6. HERV-W ENV Increases the Expression of Synaptic Proteins in DA Neurons

**Figure 5:**
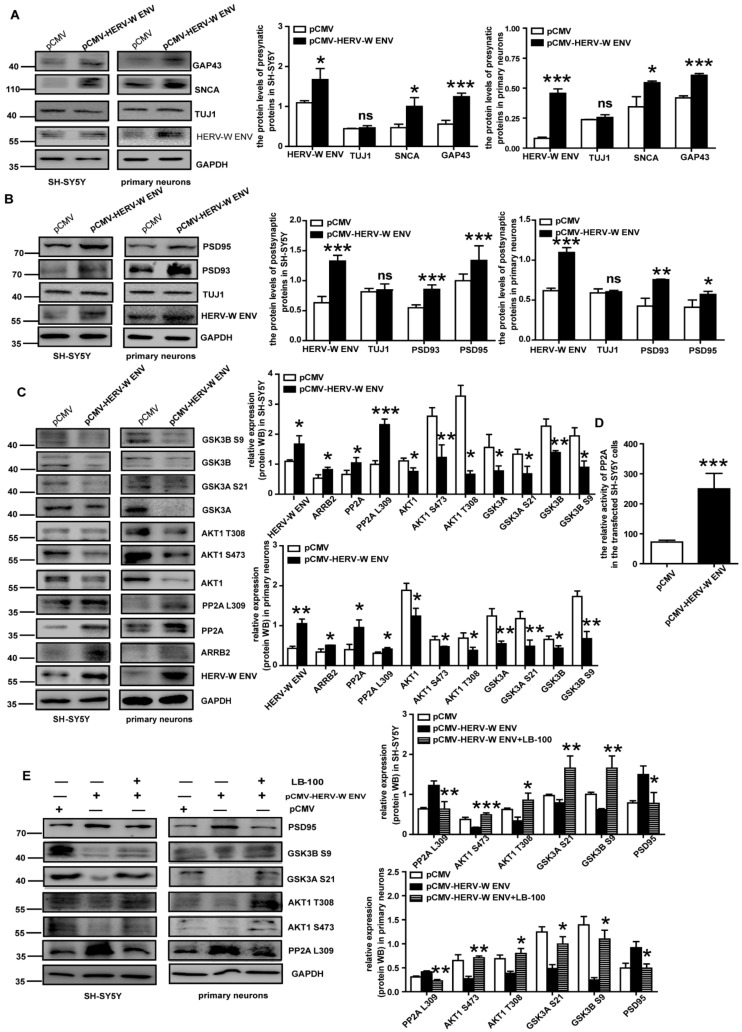
HERV-W ENV promotes synaptic proteins and regulates the PP2A/AKT1/GSK3 signal pathway: (**A**,**B**) representative Western blots for the presynaptic and postsynaptic proteins, GAPDH, and TUJ1 in the SH-SY5Y cells and the primary neurons. (**C**) Overexpression of HERV-W ENV in the DA neurons increased PP2A and AKT1 and regulated the downstream protein. (**D**) PP2A activity detected by biochemical kit assay increased in the *pCMV-HERV-W ENV* group in SH-SY5Y cells. (**E**) The PP2A was downregulated and its downstream proteins were upregulated after using LB-100 (inhibitor of PP2A) in the DA neurons. ns *p* > 0.05, * *p* < 0.05, ** *p* < 0.01, *** *p* < 0.001.

Synaptic plasticity is central to understanding the mechanisms of learning and memory. Many proteins in both presynaptic and postsynaptic membranes are involved in synaptic plasticity [49]. Representative presynaptic proteins, including SNAP23 (synaptosome associated protein 23)*,* SNAP25 (synaptosome associated protein 25), SNCA (synuclein alpha), and GAP43 (growth associated protein 43)*,* are implicated in the regulation of synaptic plasticity [50,51,52]. In this research, HERV-W ENV promoted mRNA (Figure S3) and protein (Figure 5A) levels of these presynaptic proteins. Additionally, the neuronal marker TUJ1 did not change (Figure 5A).

Postsynaptic density (PSD) is a protein dense specialization attached to the postsynaptic membrane containing many neurotransmitter receptors, scaffold proteins and signal molecules. The representative proteins in PSD, including PSD93 and PSD95, are responsible for the plasticity of the postsynaptic membrane [53]. HERV-W ENV promoted the mRNA (Figure S4) and protein (Figure 5B) levels of PSD93 and PSD95 in the DA neurons. Additionally, the level of neuronal marker TUJ1 did not change (Figure 5B).

Combined with the fact that the presynaptic and postsynaptic proteins are involved in synaptic plasticity defects [49], the above results revealed that HERV-W ENV contributes to the synaptic plasticity defects by the regulation of synaptic proteins.

### 3.7. HERV-W ENV Activates PP2A/AKT1/GSK3, the Pathway of Dopaminergic Synaptic Transmission, via DRD2

DRD2 can activate the PP2A/AKT1/GSK3 signal pathway in dopaminergic neurotransmission [20]. Combined with our previous studies showing that HERV-W ENV could directly interact with DRD2 and potentiate the expression of DRD2, we tried to investigate whether HERV-W ENV could activate the PP2A/AKT1/GSK3 pathway via DRD2 in DA neurons.

We found that HERV-W ENV elevated the levels of total PP2A and PP2A (Leu 309) methylation (Figure 5C) and enhanced PP2A activity (Figure 5D). The expression of ARRB2, a PP2A binding protein, was also upregulated (Figure 5C). However, HERV-W ENV led to an impaired expression of phosphorylated AKT1 at Ser473 and Thr308 sites, as well as both GSK3 phosphorylation (GSK3α (Ser 21) and GSK3β (Ser9)) (Figure 5C).

The results also demonstrated that LB-100 effectively decreased expression levels of PP2A methylation (Leu309) and PSD95, which were upregulated by HERV-W ENV (Figure 5E). Meanwhile, after being treated with LB-100, the levels of p-AKT1 (Ser473, Thr308), p-GSK3α (Ser21), and p-GSK3β (Ser9) were recovered (Figure 5E). From the above, HERV-W ENV activated the PP2A/AKT1/GSK3 signal pathway in the DA neurons.

**Figure 6:**
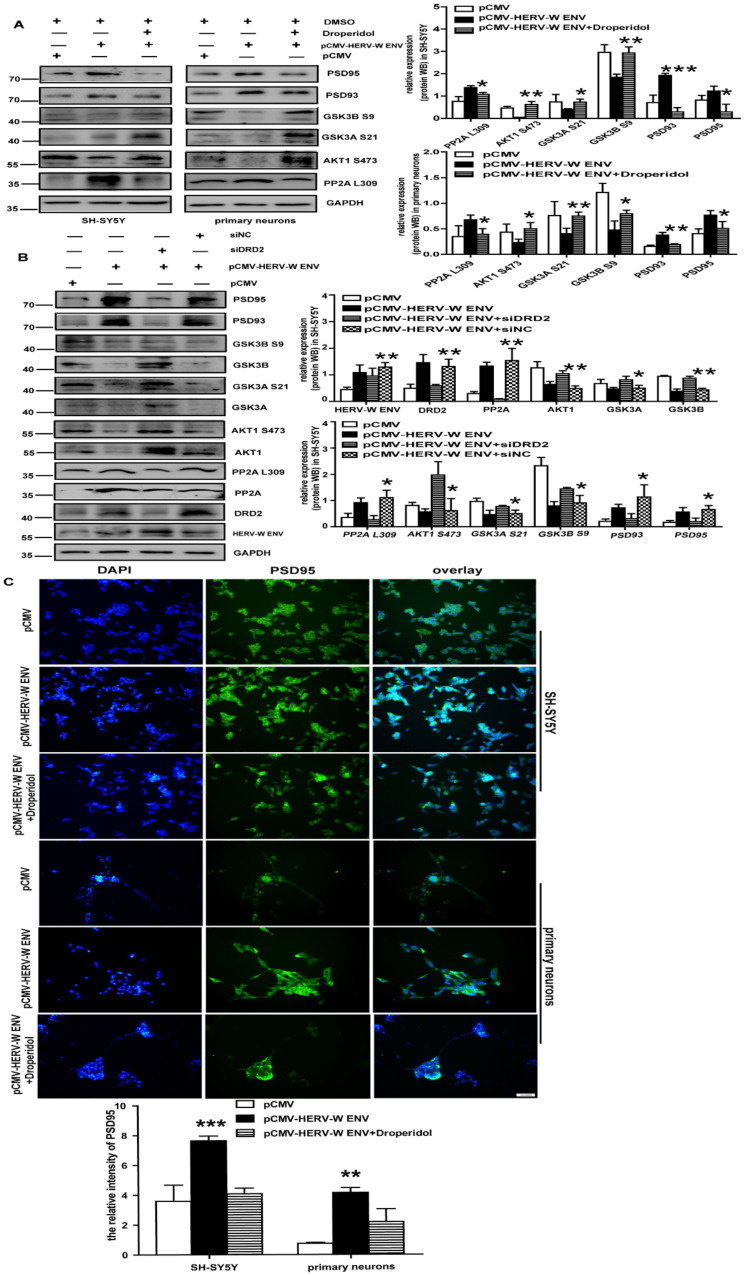
HERV-W ENV regulates the PP2A/AKT1/GSK3 signal pathway via DRD2: (**A**) the DRD2 was down-regulated and its downstream proteins were upregulated after using Droperidol (inhibitor of DRD2) in the DA neurons. (**B**) The PP2A was downregulated and its downstream proteins were regulated after down-regulating DRD2 by *DRD2 siRNA* in SH-SY5Y cells. (**C**) Immunofluorescence staining of PSD95. The immunofluorescence was higher in the HERV-W ENV overexpression group and then recovered by Droperidol by cellular immunofluorescence. Each experiment was performed three times with a representative result shown. * *p* < 0.05, ** *p* < 0.01, *** *p* < 0.001.

Droperidol was a DRD2 antagonist with a high and preferential affinity for DRD2. Data manifested that Droperidol decreased the expression levels of DRD2, methylated-PP2A (Leu309), PSD93, and PSD95, induced by HERV-W ENV (Figure 6A). As expected, protein levels of p-AKT1 (Ser473), p-GSK3α (Ser21), and p-GSK3β (Ser9) were resumed (Figure 5F). The results from the knockdown of DRD2 by short interfering *DRD2* (*si-DRD2*) (Figure 6B) were similar to Droperidol. PSD95 immunoreactivity increased after the *HERV-W ENV* transfection, but it decreased in the presence of Droperidol (Figure 6C).

We revealed that HERV-W ENV mediated the PP2A/AKT1/GSK3 signal pathway through DRD2 in the DA neurons.

### 3.8. HERV-W ENV Enhances Sodium Influx in DA Neurons

**Figure 7:**
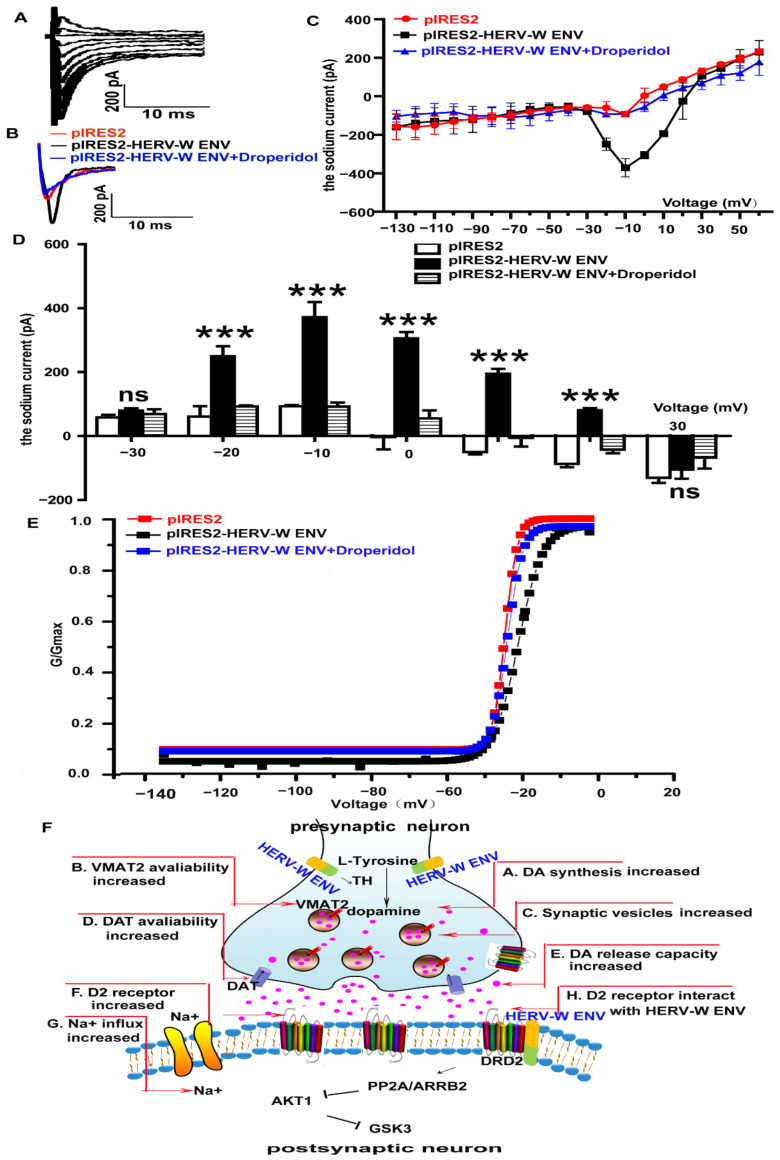
HERV-W ENV enhances the sodium influx through DRD2/PP2A/AKT1/GSK3 in the DA neurons: (**A**) a representative set of sodium current traces from SH-SY5Y cells that were elicited by depolarizing steps from −60 to 20 mV in 10-mV increments from a holding potential of −100 mV. (**B**) The sharp contrast of sodium currents in the control (the red line), HERV-W ENV (the black line), and the HERV-W ENV and Droperidol coexistence (the blue line) groups. (**C**) The average sodium currents alteration from −130 mV to 60 mV in the three groups. (**D**) The sodium currents were voltage dependent and were different in −20 mV, −10 mV, 0 mV, 10 mV, and 20 mV among the three groups. (**E**) Activation-voltage relationships obtained in the presence or absence of HERV-W ENV and Droperidol. There was a significant rightward shift of the activation-voltage relationship in the presence of HERV-W ENV (*p* < 0.01). The 1/2-activation voltage was recovered in the absence or presence of Droperidol (*p* < 0.01). (**F**) Illustration of the signaling pathway by which HERV-W ENV regulates dopaminergic system in schizophrenia. Each experiment was performed three times with a representative result shown. ns *p* > 0.05, *** *p* < 0.001.

DA modulates sodium currents in neurons [54]. Yang et al. underlines that voltage-gated Na+ channels determine neuronal excitability [55]. Sodium channels are essential in midbrain DA neurons [56]. The whole-cell patch-clamp displayed that HERV-W ENV activated sodium currents in SH-SY5Y cells (Figure 7A). The activation–voltage relationship was significantly modified (Figure 7B–D), and the 1/2-activation voltage shifted from −25.8 ± 0.8 mV to −21.8 ± 0.4 mV (*p* < 0.01) under the absence and presence of HERV-W ENV. Further investigations proved Droperidol gated sodium currents induced by HERV-W ENV (Figure 7B–D). After using Droperidol, the 1/2-activation voltage recovered from −21.8 ± 0.4 mV to −25.1 ± 0.6 mV (*p* < 0.01; Figure 7E), illustrating that HERV-W ENV could induce sodium currents via DRD2 in SH-SY5Y cells.

Our findings (Figure 7F) demonstrate that HERV-W ENV triggered the hyperactive dopaminergic system via the DRD2/PP2A/AKT1/GSK3 cascade in schizophrenia.

## 4. Discussion

HERV-W ENV is a cell-cell fusion protein essential for placental development [57]. Intriguingly, several studies have provided evidence in support of the possible pathogenic role of HERV-W ENV in schizophrenia [58]. Our previous studies outlined that HERV-W ENV can stimulate a pro-inflammatory process in schizophrenia [58,59,60,61]. In this article, our data displayed a consistency between HERV-W ENV and DRD2 in schizophrenia. Furthermore, we showed that HERV-W ENV caused structural and functional abnormalities of DA neurons through the PP2A/AKT1/GSK3 pathway via DRD2.

Several clinical investigations have reported an increased mRNA or protein expression level of HERV-W ENV in schizophrenia patients [9,10,11,12,13]. We also found that HERV-W ENV was positive (concentration ≥ 5415.1 ng/L) in 95% of onset schizophrenia patients in our article. HERV-W ENV cannot be detected in some schizophrenia patients, mainly for the following two reasons. Firstly, the expression is lower or less released in body fluids than in initial attacks. Secondly, it may correspond to a subgroup of patients with divergent etiopathogenesis from others [9].

The potential capability to diagnose neurotransmitters rapidly, sensitively, and selectively in the human serum environment is exceptionally crucial for clinical biology. As one of the critical catecholamine neurotransmitters, DA plays a significant role in the central nervous, renal, hormonal, and cardiovascular systems. Abnormal concentrations of DA are allied with neurological and physiological illnesses such as schizophrenia, Parkinson’s disease, HIV infection, and addictive behavior. Furthermore, DA is also indirectly involved in regulating blood pressure, glycogen metabolism, and functions of the immune system along with several visceral organs. Consequently, precise and periodical diagnoses of DA in physiological fluids are recognized as important therapeutic implications. Positron emission tomography (PET) imaging demonstrates that increased subcortical DA synthesis and release capacity are strongly associated with schizophrenia [19]. However, there is no report on serum DA levels in schizophrenia patients. In this study, we reported an increase in serum DA levels in schizophrenia compared with controls. Intriguingly, further analyses displayed a robust positive correlation between DA and HERV-W ENV in schizophrenia patients.

There are five types of dopamine receptors in the dopamine system, including *DRD1*, *DRD2*, *DRD3, DRD4,* and *DRD5* [37]. We once reported that HERV-W ENV increases DRD3 [10], a critical DA receptor involved in schizophrenia [62]. The DA hypothesis of schizophrenia postulates that hyperactivity of DRD2 contributes to positive symptoms of schizophrenia. However, the status of the serum of DRD2 mRNA expression in schizophrenia is controversial [39,40,41]. The regulation of DA synthesis and release is not solely regulated by presynaptic DRD2 autoreceptors on DA neurons, but postsynaptic DRD2 can participate in these functions [63]. In this article, our data displayed an elevated DRD2 and a marked consistency between HERV-W ENV and DRD2 in schizophrenia.

Our clinical analysis indicated that DA concentration was related to HERV-W ENV in schizophrenia patients. Further studies in neuronal cells found that HERV-W ENV increased DA in DA neurons. The concentration of DA depends on its synthesis, storage, release, circulation, and degradation process. TH, an enzyme important for DA synthesis, implicates schizophrenia [64]. Interestingly, HERV-W ENV increased TH, implying that HERV-W ENV might induce an increase in DA by upregulating TH. DAT is significantly higher in schizophrenia patients [40]. Analogously, HERV-W ENV strengthened the expression of DAT. These data indicated HERV-W ENV affected the synthesis and transport of DA.

In DA neurons, SVs are essential for the storage, release, and reuptake of DA. SYP, VAMP1, and VMAT2 are SV proteins involved in synaptic plasticity [65,66]. In this study, HERV-W ENV promoted them. Subsequently, SVs are recovered and recycled by endocytosis [67]. Our data showed that HERV-W ENV increased the number of SVs from the cell body toward the axon terminals. Specifically, our findings highlighted that HERV-W ENV promoted vesicle formation and transport of SVs to increase DA release.

The action of DA occurs via DA receptors. Here, we found that HERV-W ENV increased DRD2 via its promoter. Co-IP assays showed a direct interaction between DRD2 and HERV-W ENV. Moreover, there was an apparent colocalization between them. Taken together, these results suggest that HERV-W ENV might have distinct functions in the dopaminergic system.

DA neurons consist of a presynaptic membrane, synaptic cleft, and postsynaptic membrane. SNAP23, SNAP25, SNCA, and GAP43, present in the presynaptic membrane, play essential roles in neurotransmitter release and plasticity [51,52,68] and are associated with schizophrenia [47,69,70]. We found that HERV-W ENV significantly induced an increase in them. After being released into the synaptic cleft, DA binds to receptors located on the postsynaptic membrane. PSD93 and PSD95, located in postsynaptic membrane, are linked to the neuropathology of schizophrenia [71]. Our results revealed that HERV-W ENV upregulated the expression of PSD93 and PSD95. In a word, HERV-W ENV might play a pivotal role in neurotransmitter release and synaptic proteins, identifying a new functional role of HERV-W ENV in DA neurons.

DRD2, one of the essential receptors in the DA system, can inhibit the activity of AKT through a PP2A-dependent AKT signal pathway. Several antipsychotic drugs (DRD2 antagonists) can activate AKT and increase the inhibitory phosphorylation of GSK3 in the rodent brain. Serine/threonine phosphatases play a crucial role in synaptic plasticity. Some researchers have reported altered serine/threonine kinase activity in schizophrenia. Though the bioinformatics of microarrays were limited based only on *p* < 0.05 and not the more rigorous statistical threshold of the false discovery rate (FDR), GO and KEGG analyses also revealed that these 255 DEGs were mainly involved in protein serine/threonine kinase activity and the Hippo pathway. PP2A, a widely conserved protein serine/threonine phosphatase (PSP), is a negative regulator of the Hippo pathway. Single-nucleotide polymorphisms (SNPs) of PP2A are involved in schizophrenia [72]. Interestingly, we found that HERV-W ENV could lead to the methylation of PP2A (Leu 309). The increase in PP2A methylation and activity leads to a decrease in AKT1 phosphorylation (Ser473, Thr308), which means the inactivation of AKT1. Reduced AKT1 protein levels are also reported in schizophrenia patients [22]. Inactivation of AKT1 usually leads to reduced expression of phosphorylated GSK3. Meanwhile, the impairment of the AKT/GSK3 pathway is directly associated with schizophrenia [22]. Extensive studies have indicated decreased levels of GSK3α and GSK3β total protein in the lymphocytes of schizophrenia [22]. Interestingly, our study showed the dephosphorylation of GSK3α (Ser21) and GSK3β (Ser9) through PP2A/AKT1 in the DA neurons induced by HERV-W ENV. On the contrary, bioinformatics revealed that the mRNA levels of GSK3β were higher in schizophrenia. Inconsistencies between changes in GSK3β RNA and protein expression may be due to its alternative splicing [73]. GSK3β expression varies widely in different cell types [74]. There are also increasing shreds of evidence that GSK3β plays diverse roles in various kinds of nerve cells [75,76]. In our previous study, HERV-W ENV enhanced the phosphorylation of GSK3β (Ser9) in glial cells [77]. This study found that HERV-W ENV played a controversial role in regulating the phosphorylation of GSK3β in neuronal cells, implying that GSK3β might play diverse roles in different cells. Therefore, our data showed that HERV-W ENV activated the ARRB2/PP2A/AKT1/GSK3 pathway.

The DRD2-mediated AKT/GSK3 signal seems to have significant effects on dopaminergic transmission. Droperidol is a small molecule that selectively inhibits DRD2. In this study, we found that Droperidol inhibited the PP2A/AKT1/GSK3 pathway and was induced by HERV-W ENV in DA neurons. Interestingly, the protein levels of PSD93 and PSD95 recovered in DA neurons after treatment with Droperidol, *siDRD2*, and LB-100. These data demonstrated that HERV-W ENV might activate the PP2A/AKT1/GSK3 signal pathway through DRD2 in the dopaminergic system.

Our study shows that HERV-W ENV regulates brain-derived neurotrophic factor (BDNF) [10], the primary function of which is to regulate synapses [49] via the phosphorylation of GSK3β at Ser9 [77]. Additionally, HERV-W ENV can also elevate intracellular calcium influx [78] and activate small conductance Ca^2+^-activated K^+^ channel protein 3 (SK3) channels [79], which both are necessary for neurotransmitter release and synaptic plasticity [80]. These findings suggest that HERV-W ENV likely plays a vital role in dysfunctional neural plasticity in the pathophysiology of schizophrenia. Repeated activation of the dopaminergic system in the midbrain leads to persistent behavioral changes, accompanied by the neural plasticity pattern of nucleus accumbens (NAc).

Meanwhile, evidence suggests that GSK3 activation promotes neuronal differentiation, whereas inhibition of GSK3β induces the formation of multiple axon-like neurites in hippocampal neurons [81]. Coupled with our discovery that HERV-W ENV altered the expression or activation of GSK3β, we proposed that HERV-W ENV might play a critical role in DA neurons. DA neurons display a range of activity modes that vary in degrees of burst firing. Calcium accumulation can promote the removal of synapses mediated by phagocytes in cortical neuroinflammation [82]. DA neuronal bursting, associated with DA release, is driven by sodium channels. Sodium channels are the primary ion channels and critical targets for neuronal excitability and neuromodulation in DA neurons [55]. Meanwhile, biophysical properties of sodium voltage in DA neurons differ from those of GABAergic neurons [55]. Sodium ions are essential for spike generation in DA neurons. The sodium current is used to determine whether cells electrophysiologically resemble mature DA neurons [83]. Moreover, the sodium channel, voltage-gated type II α subunit gene *SCN2A*, has been shown to exhibit mutations in schizophrenia [84]. Bioinformatics predicted that DEGs mainly focused on the sodium ion transmembrane transport, and patch-clamp analysis indicated that HERV-W ENV enhanced sodium influx through DRD2.

HERV-W ENV is related to the pathogenesis of many diseases, and clinical treatment of MSRV has been carried out. GNbAC1, an IgG4 monoclonal antibody, has been developed to specifically target the MSRV-specific epitope and to neutralize the effect of envelop protein both in vitro [85] and in vivo [86]. GNbAC1 is currently in clinical development for MS. Of note, in relapsing MS patients, a phase 2b clinical trial using GNbAC1 was conducted (CHANGE-MS, NCT02782858) [87]. GNbAC1 is also now being tested in patients who have type 1 diabetes (T1D) and is in phase 2a clinical trials [86]. Considering GNbAC1 has been favorable in clinical trials of patients with MS and type 1 diabetes [88,89], HERV-W ENV may also present a promising potential therapeutic target for schizophrenia.

## 5. Conclusions

Our studies reported a robust increase in DA, DRD2, and HERV-W ENV in schizophrenia and displayed a positive correlation and marked consistency between DRD2 and HERV-W ENV. Studies in vitro indicated that HERV-W ENV increased DA concentrations by promoting DA synthesis and transport, and HERV-W ENV activated the dopaminergic system by upregulating the DRD2 and synaptic proteins. Furthermore, HERV-W ENV could enhance the sodium currents and the hyperactive PP2A/AKT1/GSK3 pathway through DRD2 by colocalizing and interacting with it. These findings provide new evidence for the DA hypothesis and open a new window towards the pathogenesis of schizophrenia. In summary, HERV-W ENV dysfunction is a potential pathogenic factor in schizophrenia and can be a new marker and therapeutic target for the diagnosis and treatment of schizophrenia.

## Figures and Tables

**Table 1:** The consistence of HERV-W ENV and DA concentration in schizophrenia patients and healthy controls.

		HERV-W ENV (+)	HERV-W ENV (−)	Consistency Ratio
Schizophrenia patients	DA (+)	29	18	63.16%
	DA (−)	3	7	
Healthy controls	DA (+)	6	17	45.59%
	DA (−)	14	31	

**Table 2:** The Spearman analysis between HERV-W ENV and DA in schizophrenia patients.

		HERV-W ENV	DA
HERV-W ENV	Correlation coefficient	1.000	0.396 **
	Sig.	−	0.002
	n	57	57
DA	Correlation coefficient	0.396 **	1.000
	Sig.	0.002	−
	n	57	57

**. *p* < 0.01.

## Data Availability

All data is available from the corresponding author upon request.

## Supplementary Materials

The following supporting information can be downloaded at: https://www.mdpi.com/article/10.3390/v14010145/s1, Figure S1: Immunofluorescence staining of rat primary dopaminergic neurons, Table S1: human primer sequences, Table S2. rat primer sequences, Table S3: antibodies and the dilutions, Table S4: gene ontology analysis of differentially expressed genes in schizophrenia, Table S5: KEGG pathway analysis of differentially expressed genes in schizophrenia, Table S6: comparison of demographic and clinical characteristics between the normal/onset schizophrenia (SCZ) groups in Chinese, Table S7: the concentration of DA in control and SCZ, Table S8: the concentration of DRD1 in control and SCZ, Table S9: the concentration of DRD2 in control and SCZ, Table S10: the concentration of HERV-W ENV in control and SCZ, Table S11: the concentration of dopamine in the HERV-W ENV (−) and HERV-W ENV (+) SCZ patients, Table S12: the concentration of DRD2 in the HERV-W ENV (−) and HERV-W ENV (+) SCZ patients, Figure S2: HERV-W ENV increases the mRNA levels of vesicle-related proteins and the promoter activity of VMAT2, Figure S3: HERV-W ENV increases the mRNA levels of presynaptic proteins, Figure S4: HERV-W ENV increases the mRNA levels of postsynaptic proteins.
