# Supplementary material for: HERV-W Envelope Triggers Abnormal Dopaminergic Neuron Process through DRD2/PP2A/AKT1/GSK3 for Schizophrenia Risk
Source: Viruses. 2022 Jan 14;14(1):145. doi: 10.3390/v14010145 (PMC8777930; doi:10.3390/v14010145)
Supplement: Supplementary file 1 [file viruses-14-00145-s001.zip › viruses-1526134-supplementary.pdf]

Supplementary Materials

HERV-W Envelope Triggers Abnormal Dopaminergic Neuron Process through DRD2/PP2A/AKT1/GSK3 for Schizophrenia Risk

Qiujin Yan [1], Xiulin Wu [1], Ping Zhou [1], Yan Zhou [1], Xuhang Li [1], Zhongchun Liu [2], Huawei Tan [2], Wei Yao [1], Yaru Xia [1], Fan Zhu [1,3]*

[1] State Key Laboratory of Virology, Department of Medical Microbiology, School of Basic Medical Sciences, Wuhan University, Wuhan 430071, P.R. China

[2] Department of Psychiatry, Renmin Hospital of Wuhan University, Wuhan 430061, P.R. China

[3] Hubei Province Key Laboratory of Allergy & Immunology, Wuhan University, Wuhan 430071, P.R. China

* Correspondence: fanzhu@whu.edu.cn

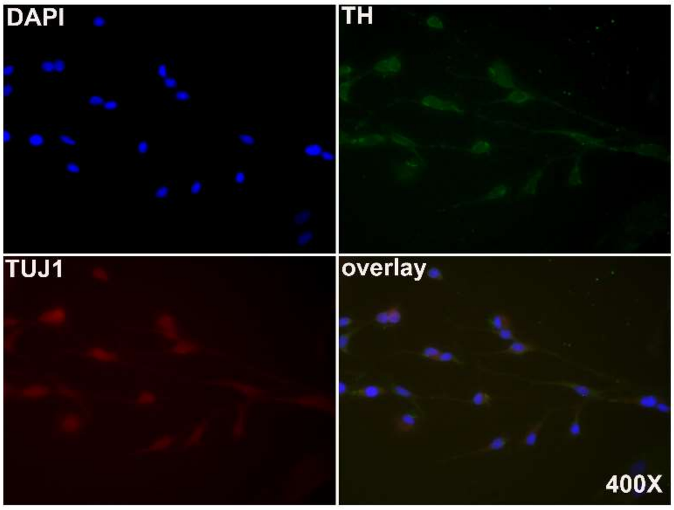

Supplementary Figure S1. Immunofluorescence staining of rat primary dopaminergic neurons. The primary dopaminergic neurons were isolated from the rat midbrain VTA (in the middle of midbrain) and cultured in vitro. The primary neurons were identified by immunofluorescence with the double-staining with TH and TUJ1 antibodies. Magnification, 400×.

Supplementary Table S1. Human primer sequences.

Name	Sequence	Length（bp）
HERV-W ENV-F	CCATGCCGCTGTATGACCAG	109
HERV-W ENV-R	GGGTTCCCTTAGAAAGACTCCT	
SNAP23-F	TGGAAGAGAACCTGACTCAAGT	120
SNAP23-R	CAGCCTTGTCTGTGATTCGTTT	
SNAP25-F	TCGTGTAGTGGACGAACGG	158
SNAP25-R	TCTCATTGCCCATATCCAGGG	
SYN1-F	AGCTCAACAAATCCCAGTCTCT	103
SYN1-R	CGGATGGTCTCAGCTTTCAC	
SNCA-F	TGTAGGCTCCAAAACCAAGG	139
SNCA-R	TGCTCCCTCCACTGTCTTCT	
GAP43-F	GGCCGCAACCAAAATTCAGG	167
GAP43-R	CGGCAGTAGTGGTGCCTTC	
TH-F	CGACCCTGACCTGGACTTGGA	142
TH-R	GGCAATCTCCTCGGCGGTGT	
DAT-F	CCATACTGAAAGGTGTGGGC	146
DAT-R	CCAGGAGTTGTTGCAGTGGA	
SYP-F	GAAGGTGCTGCAATGGGTCT	143
SYP-R	GCCTGAAGGGGTACTCGAAC	
VAMP1-F	ACATGACCAGTAACAGACGACT	76
VAMP1-R	ACGTTCACACGTATGATGTCC	
VMAT2-F	TGGACAACATGCTGCTCACTG	137
VMAT2-R	GCTCTGGAAGCTGTCTGAGATGG	
PSD93-F	GCCGGTGATTATCCTGGGG	108

PSD93-R	CGCTTTGGCCTCGTAGTATGA	
PSD95-F	CTCGATGTCTCGGCCAATG	120
PSD95-R	CTCTGTGATCCGCTTGTTAATCTC	
DRD2-F	CCCCGCCAAACCAGAGAAG	89
DRD2-R	TTTTGCCATTGGGCATGGTCT	
GAPDH-F	CTGGGCTACACTGAGCACC	101
GAPDH-R	AAGTGGTCGTTGAGGGCAATG	

Supplementary Table S2. Rat primer sequences.

Primer	Sequence	Length（bp）
SNAP23-F	GGGGAACAACTAAATCG	183
SNAP23-R	GCTGTCTCCACCATCAC	
SNAP25-F	TGGCTGATGAGTCCCT	109
SNAP25-R	AGTTGTTCGCCTTGCT	
SYN1-F	CCTCGCTGTCTAACGC	164
SYN1-R	CCCTTTGAAGTATTTTGC	
SNCA-F	CGTCCTCTATGTAGGTTCC	85
SNCA-R	TGTCACTTGTTCTTTGGTC	
GAP43-F	AGAAGGGCGAAGGGGATG	100
GAP43-R	TTGGAGGACGGCGAGTT	
TH-F	CCTTCCAGTACAAGCACGGT	109
TH-R	TGGGTAGCATAGAGGCCCTT	
DAT-F	CCTATGGAAGGGAGTAAA	159
DAT-R	TCACAGAGTCGGTAGAAGT	
SYP-F	GTGCCAACAAGACGGAG	99
SYP-R	CTTTGACGCAGGAGGGT	
VAMP1-F	AGACGATTACAGCAAACC	106
VAMP1-R	CATCCAACTCTGACAACTT	
VMAT2-F	CTCCTGCTCATCCGTG	208
VMAT2-R	AATAGCCCCATCCAAG	
PSD93-F	GCCAAGAAAACCTATGACCG	155
PSD93-R	TTGAGGGAATCCAGATGAAA	
PSD95-F	CAACACGGACACCCTA	102
PSD95-R	CCTGAGTTACCCCTTTC	
DRD2-F	TGGTGGGTGAGTGGAA	108
DRD2-R	TGTCAATGCTGATGGC	
GAPDH-F	GCATCTTCTTGTGCAGTGCC	105
GAPDH-R	GAGAAGGCAGCCCTGGTAAC	

Supplementary Table S3. Antibodies and the dilutions.

Proteins	Brands	Catalogs	Dilutions
TH	Abcam	ab137869	1:5000 (WB), 1:100 (IF)
TUJ1	Abcam	ab78078	1:150 (IF)

GAPDH	Abcam	ab8245	1:10000 (WB)
HERV-W ENV	Abcam	ab179693	1:1000 (WB), 1:100(IP)
DRD2	Abcam	ab85367	1:1000 (WB), 1:200 (IP)
GAP43	Abcam	ab75810	1:100000 (WB)
DAT	Abcam	ab111468	1:1000 (WB)
SYP	Abcam	ab14692	1:1000 (WB), 1:100 (IF)
VAMP1	Abcam	ab151712	1:5000 (WB)
VMAT2	Abcam	ab70808	1:1000 (WB)
PSD93	Abcam	ab94588	1:1000 (WB)
PSD95	Abcam	ab18258	1:1000 (WB), 1:200 (IF)
ARRB2	Abcam	ab206972	1:1000 (WB)
PP2A	Abcam	ab32104	1:5000 (WB)
AKT1	Abcam	ab227100	1:2000 (WB)
AKT1 p-S473	Abcam	ab81283	1:5000 (WB)
AKT1 p- T308	Abcam	ab105731	1:2000 (WB)
GSK3A	Abcam	ab40870	1:5000 (WB)
GSK3A p- S21	Abcam	ab131112	1:1000 (WB)
GSK3B	Abcam	ab131356	1:1000 (WB)
GSK3B p- S9	Abcam	ab131097	1:1000 (WB)

Supplementary Table S4. Gene ontology analysis of differentially expressed genes in schizophrenia.

Category	Term	Count	%	p-Value	FDR
GOTERM_BP_DIRECT	inflammatory response	21	0.0	3.1E-3	9.7E-1
GOTERM_BP_DIRECT	protein phosphorylation	22	0.0	1.1E-2	9.6E-1
GOTERM_BP_DIRECT	cell adhesion	22	0.0	1.2E-2	9.4E-1
GOTERM_BP_DIRECT	immune response	20	0.0	1.9E-2	9.7E-1
GOTERM_BP_DIRECT	negative regulation of cell proliferation	18	0.0	3.8E-2	9.8E-1
GOTERM_BP_DIRECT	**sodium ion transmembrane transport***	6	0.0	4.6E-2	9.9E-1
GOTERM_CC_DIRECT	Protein complex	22	0.0	3.3E-3	7.1E-1
GOTERM_CC_DIRECT	Cell surface	26	0.0	5.3E-3	6.3E-1
GOTERM_CC_DIRECT	integral component of plasma membrane	52	0.1	1.5E-2	7.5E-1
GOTERM_MF_DIRECT	Mitogen-activated protein kinase binding	5	0.0	9.5E-4	5.0E-1
GOTERM_MF_DIRECT	**Protein serine/threonine kinase activity**	21	0.0	3.2E-3	6.8E-1
GOTERM_MF_DIRECT	**Protein kinase activity**	20	0.0	4.2E-3	5.4E-1

GOTERM_MF_DIRECT	ATP binding	56	0.1	1.2E-2	8.1E-1
GOTERM_MF_DIRECT	Transcription regulatory region DNA binding	13	0.0	1.3E-2	8.0E-1
GOTERM_MF_DIRECT	Calcium ion binding	29	0.0	3.3E-2	8.9E-1

Note: *the terms labeled bold are vital points in this study.

Supplementary Table S5. KEGG pathway analysis of differentially expressed genes in schizophrenia.

Category	Term	Count	%	p-Value	FDR
KEGG_PATHWAY	**Hippo signaling pathway***	11	0.0	9.4E-3	8.8E-1
KEGG_PATHWAY	TNF signaling pathway	9	0.0	9.8E-3	6.7E-1
KEGG_PATHWAY	Adipocytokine signaling pathway	6	0.0	4.5E-2	9.7E-1
KEGG_PATHWAY	Adherens junction	6	0.0	4.8E-2	9.4E-1

Note: *the term labeled bold is regulated by Protein kinase, protein phosphorylation and protein serine/threonine kinase.

Supplementary Table S6. Comparison of demographic and clinical characteristics between the normal /onset schizophrenia (SCZ) groups in Chinese.

Group	Gender（n）		Age ($\bar{x} \pm S$)	BMI ($\bar{x} \pm S$)	Education years（n）					Smoking（n）	
	Male	Female			3	5	9	12	16	Yes	No
SCZ patients (57)	24	33	42.16±12.78	21.54±2.66	3	4	12	24	14	20	37
Healthy controls (68)	31	37	43.26±11.62	21.39±3.48	3	8	17	23	17	26	42
t/X²	0.153		0.507	0.284	1.551					0.132	
P value	0.417		0.613	0.777	0.818					0.852	

Supplementary Table S7. The concentration of DA in control and SCZ.

Groups	Mean	Median	Range	Minimum	Maximum	Std. Deviation	Skewness	Std. Error Skewness
Control (n=68)	3990.7	3516.1	5607.8	1996.6	7604.5	1296.6	0.71	0.7
SCZ (n=57)	15606.3	14776.3	32155.6	3931.5	36087.1	7023.9	0.41	0.4

Supplementary Table S8. The concentration of DRD1 in control and SCZ.

Groups	Mean	Median	Range	Minimum	Maximum	Std. Deviation	Skewness	Std. Error Skewness
Control (n=68)	17.96	16.76	63.06	2.13	65.19	8.78	3.43	3.35
SCZ (n=57)	16.88	15.91	50.14	2.97	53.10	7.13	2.41	2.34

Supplementary Table S9. The concentration of DRD2 in control and SCZ.

Groups	Mean	Median	Range	Minimum	Maximum	Std. Deviation	Skewness	Std. Error Skewness
Control (n=68)	1294.2	1311.1	3307.7	122.2	3429.8	661.5	0.28	0.27
SCZ (n=57)	8825.1	3732.4	79572.7	429.2	80001.9	14137.2	3.4	3.3

Supplementary Table S10. The concentration of HERV-W ENV in control and SCZ.

Groups	Mean	Median	Range	Minimum	Maximum	Std. Deviation	Skewness	Std. Error Skewness
Control (n=68)	4538.1	3950.6	26909	522.5	27431.5	3623.3	3.8	3.7
SCZ (n=57)	15246.8	8786.2	144777.9	1658	146435.9	24114.2	3.7	3.6

Supplementary Table S11. The concentration of dopamine in the HERV-W ENV (-) and HERV-W ENV (+) SCZ patients.

Groups	Mean	Median	Range	Minimum	Maximum	Std. Deviation	Skewness	Std. Error Skewness
HERV-W ENV (-)	12585.5	10389.2	21735.6	4376.1	26111.7	5950.1	0.8	0.8
HERV-W ENV (+)	17966.3	18654	32155.6	3931.5	36087.1	6893.5	0.1	0.1

Supplementary Table S12. The concentration of DRD2 in the HERV-W ENV (-) and HERV-W ENV (+) SCZ patients.

Groups	Mean	Median	Range	Minimum	Maximum	Std. Deviation	Skewness	Std. Error Skewness
HERV-W ENV (-)	1872.5	1944.6	2177.6	429.2	2606.7	447.7	1.2	1.1
HERV-W ENV (+)	14256.9	8710.4	77555.1	2446.8	80001.9	16987.5	2.6	2.5

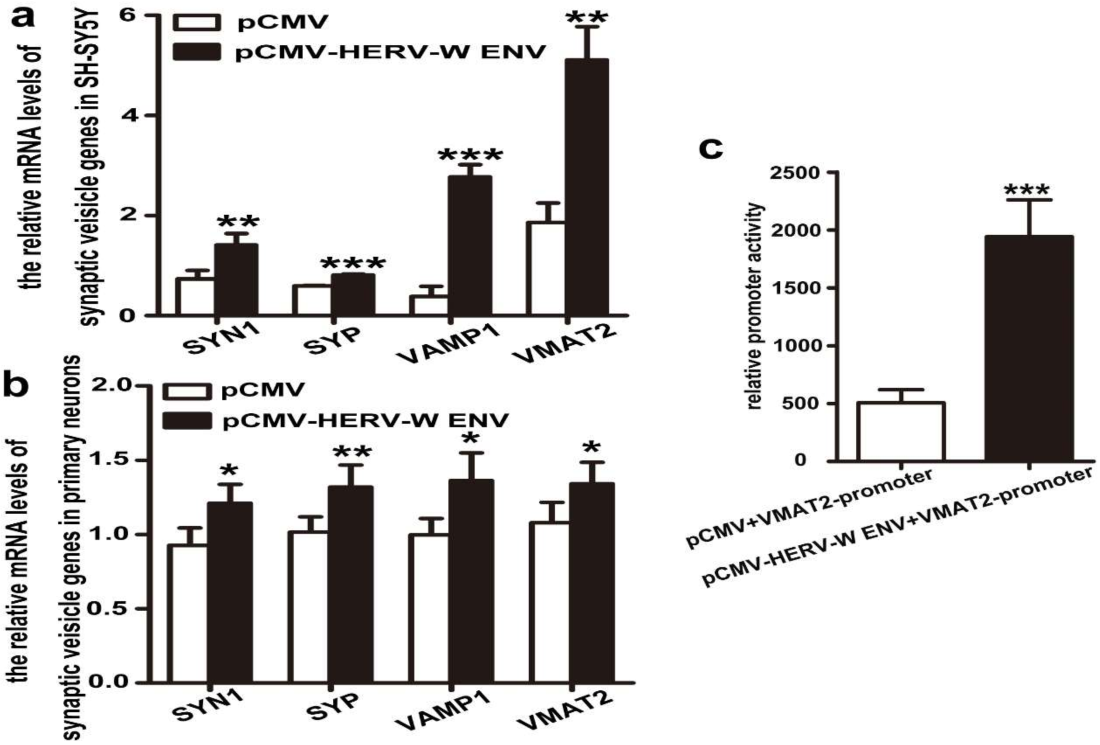

Supplementary Figure S2. HERV-W ENV increases the mRNA levels of vesicle-related proteins and the promoter activity of *VMAT2*. (a) Real-time PCR for the synaptic vesicle-related proteins in SH-SY5Y cells. (b) Real-time PCR for the synaptic vesicle-related proteins in the primary dopaminergic neurons. (c) Luciferase assays of *pGL3-VMAT2* promoter co-transfected with *pCMV-HERV-W ENV* or control vector in SH-SY5Y cells.

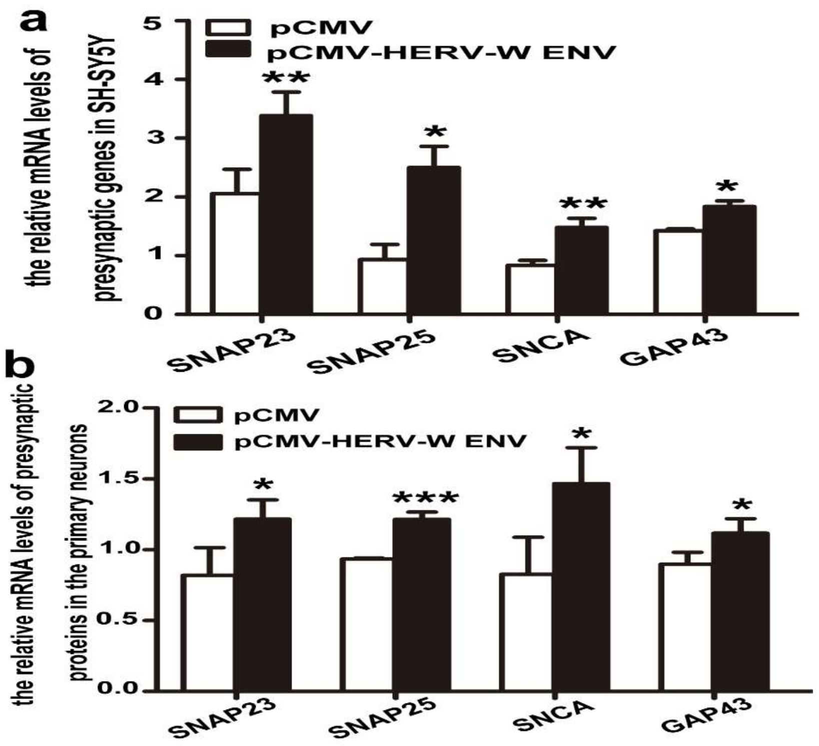

Supplementary Figure S3. HERV-W ENV increases the mRNA levels of presynaptic proteins. (a) Real-time PCR for the presynaptic proteins in SH-SY5Y cells. (b) Real-time PCR for the presynaptic proteins in the primary dopaminergic neurons.

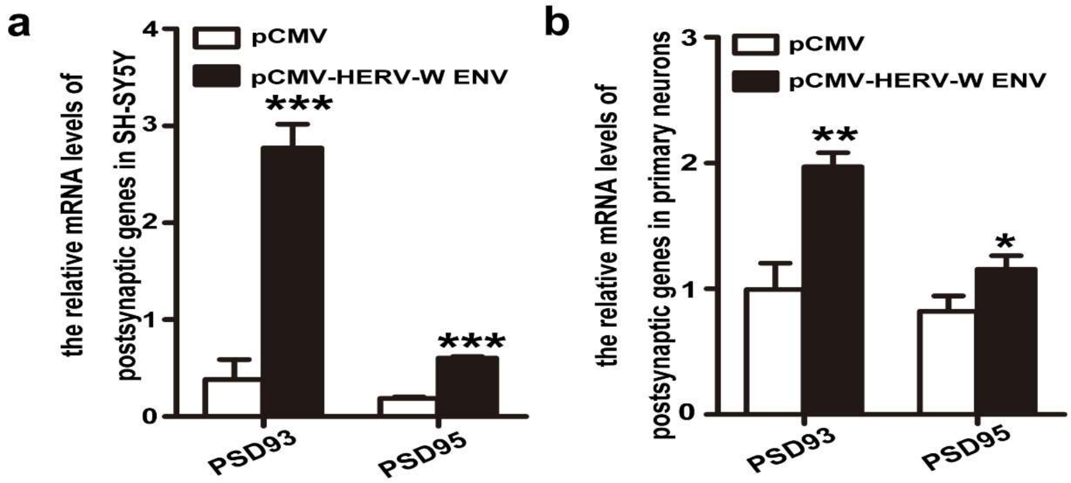

Supplementary Figure S4. HERV-W ENV increases the mRNA levels of postsynaptic proteins. (a) Real-time PCR for the postsynaptic proteins in the SH-SY5Y cells. (b) Real-time PCR for the postsynaptic proteins in the primary dopaminergic neurons.
